# The Dark Triad traits in the South African workplace: moderators of career interests and success

**DOI:** 10.3389/fpsyg.2025.1588364

**Published:** 2025-06-04

**Authors:** Denise Oldewage, Petronella Jonck

**Affiliations:** Department of Industrial Psychology and People Management, University of Johannesburg, Johannesburg, South Africa

**Keywords:** Dark Triad traits, narcissism, psychopathy, Machiavellianism, career interests, career success, moderation analysis

## Abstract

**Introduction:**

Numerous studies have been conducted on the nexus between the Dark Triad personality traits and the influence thereof within the context of work. However, a paucity of studies underscores the interaction between these traits with career interests and success, particularly within the South African context. This study aimed to investigate the direct and indirect effect of the Dark Triad traits on career interest and career success.

**Methods:**

A quantitative approach utilizing a cross-sectional design collected 406 responses from economically active participants using convenience sampling. The structured self- reported survey included the South African Career Interest Inventory (SACII-Short), The Dark Triad Dirty Dozen (DTDD) and the Subjective Career Success Inventory (SCSI). Structural equation modeling (SEM) and moderation analysis were performed to examine the data.

**Results:**

Psychopathy had a statistically significant negative effect on both career interests and career success. Contrarily, narcissism did not have a statistically significant impact on either career interest or career success. Machiavellianism had a detrimental effect on career success but not career interest. The moderation analysis supported the hypothesis that psychopathy moderates the relationship between investigative career interest and career success negatively, underscoring the disruptive nature of this trait on career outcomes.

**Discussion:**

Organizations should practice caution when hiring or promoting individuals with Dark Triad traits, particularly in positions requiring collaboration, trust and long-term success. This could be achieved by integrating personality assessments such as the DTDD into leadership development, talent development and HR policies interventions to reduce the negative consequences of the mentioned attributes to career success.

## 1 Introduction

Organizations are continuously searching for ways to achieve a competitive advantage, especially with the acceleration of technological advancements (Lyons, 2019). A method used to achieve the forestated is by using personality assessments since personality traits have been proven to predict person-job fit (Niessen et al., 2016; Peral, 2019), increase motivation (Chirumbolo, 2017; Nuckcheddy, 2018) and improve performance (Jonason et al., 2014; Schneider et al., 2017). While most assessments focus on the positive aspects of personality, interest in the darker side of personality has garnered increased attention (O’Boyle et al., 2012; Jonason et al., 2019; Schneider et al., 2017). The Dark Triad traits, a term initially coined by Paulhus and Williams (2002), is made up of the less favorable traits of narcissism, Machiavellianism and psychopathy – all of which share similar underlying themes of manipulation, deceit, apathy, and coldness (Lyons, 2019). Individuals scoring high on the Dark Triad traits are known to be self-serving and ruthless in putting internalized needs and interests foremost toward achieving success, often employing unethical and exploitative tactics (Furnham et al., 2014).

Expanding on the notion of matching employees with suitable jobs, which is essential for organizational productivity (Nuckcheddy, 2018), a positive organizational culture (Lyons, 2019) and employee satisfaction as well as motivation (Volodina et al., 2015), it is important to acknowledge how personality traits influence the person-job alignment. Person-job fit can be achieved by matching an individual’s personality traits and career interests (Morgan and de Bruin, 2019; Schneider et al., 2017). As such, Holland (1997) states that person-job fit is pivotal in shaping individuals’ career choices and subsequent career paths based on interests and personality traits. Individuals aligned with innate career interests are more likely to be productive and have increased motivation, job satisfaction (Kristof-Brown et al., 2005), and job fulfilment (Stoll et al., 2020; Su et al., 2015). Therefore, person-job fit is likely to result in long- term career growth and satisfaction (Volodina et al., 2015) based on the assumption that the forestated would increase the probability of financial stability, career advancement, and ultimately achieving career success (Holland, 1997; Morgan and de Bruin, 2019; Nauta, 2010).

Career success is a multi-dimensional concept (Yu et al., 2022) that measures both objective (extrinsic) success in the form of salary, rewards and promotions received (Rostyslav and Vsevolod, 2024; Shockley et al., 2016), as well as subjective (intrinsic) success, referring to individual perceptions, job satisfaction, meaningfulness and work-life balance (Koekemoer and Crafford, 2019; Shockley et al., 2016; Wille et al., 2012). A subjective view of career success is of particular interest to the research reported on in this study. Career success is the “accumulation of positive work and psychological outcomes from one’s work experiences” over a career span (Seibert and Kraimer, 2001, p. 2). Both objective and subjective career success has been shown to predict increased job satisfaction (Audibert et al., 2022; Olson and Shultz, 2013), motivation (Barrick et al., 2002), commitment (Briscoe et al., 2021) and productivity (Guenole, 2014). Therefore, career success positively contributes to employee and organizational performance (Feldman and Ng, 2007; Spurk et al., 2018). Career success has been linked to career interest (Hirschi et al., 2017; Ng and Feldman, 2014). Extant research underscoring the relationship between career interests and career success has consistently shown that individuals following a career path best suited to innate personality traits and areas of interest are likelier to report job satisfaction and fulfilment through intrinsic and extrinsic motivation (Earl et al., 2019), all the while displaying increased commitment levels, productivity and overall work performance (Akosah-Twumasi et al., 2018; Earl et al., 2019; Ryu and Jeong, 2021).

Notwithstanding the positive outcomes associated with career success, a more sinister reality exists in the pursuit thereof, involving dishonesty and morally questionable behavior (Baloch et al., 2017). Burgeoning literature indicates that a group of ‘darker’ personality traits (viz., the Dark Triad traits), influence career-related decision-making and unethical means to attain career success (Lyons, 2019). The presence of the Dark Triad traits could create tension in employee relationships ascribed to the ill treatment of others within the organization, resulting in conflict and potentially disrupting organizational performance (Peral, 2019).

## 2 Problem statement

Organizations are realizing the pivotal role of employees as the organization’s most important resource (Gabčanová, 2011). As such, greater emphasis is placed on people management and improving performance, satisfaction and success. However, Smit (2020) reflecting on previous research by Guenole (2014) emphasizes the need to expand the scope beyond the positive aspects of behavior and investigate counterproductive personality traits, such as the Dark Triad traits. The corpus of knowledge underscores how the Dark Triad traits shape individual behaviors and personalities as well as individual performance in specific job roles (Dries et al., 2008; Feldman and Ng, 2007; Gruys, 1999; O’Boyle et al., 2012; Paulhus and Williams, 2002; Vardi and Weitz, 2016). However, a lacuna in the corpus of knowledge relates to the interplay of the mentioned constructs as well as the buffering or moderating effect the Dark Triad traits have on career interests as a predictor of career success within the South African context. As such, Cohen and Özsoy (2021) expound that a paucity of studies investigates the association between the Dark Triad traits and career success and, by extension, career interest. It should be noted that the research reported on assumes that each trait independently affects career interest and success but does not investigate whether combinations of traits (e.g., Machiavellianism and psychopathy) might have synergistic or moderating effects.

## 3 Literature review

### 3.1 Dark Triad traits

The Dark Triad traits consisting of narcissism, Machiavellianism and psychopathy were coined by seminal authors Paulhus and Williams (2002) based on the assumption that the darker sides of personality should be viewed as an entity. The forestated would provide a more holistic and well-rounded view of an individual’s ‘darker side’, sharing similarities (Mangera, 2019). The effects of the Dark Triad traits within the context of work can be destructive, for example between colleagues, within teams, and among customers, but also toward the organization at large (Schyns et al., 2022). *Per se*, the negative consequences of employees with Dark Triad traits in the organization, especially in upper-level management or leadership, can be dire if not addressed or adequately managed (Guenole, 2014; Spain et al., 2013). For this reason, greater attention should be paid to the undesirable side of personalities (Smit, 2020), often negated by focusing on positive psychology, attitudes and behaviors in the organization (Harms, 2022; Mackey et al., 2020). The Dark Triad traits will be further elaborated on to comprehend the potential consequences thereof.

#### 3.1.1 Narcissism

Levander et al. (2018) explains that the construct, narcissism, was first mentioned in psychology literature in 1911. Specifically, vanity and self-admiration were linked to *Narcissus*, a mythical Greek figure who became obsessed with his own image (Levander et al., 2018). Narcissism was then brought to the forefront when Freud published an essay on the topic in 1914 (Freud, 2014), discussing narcissism in the context of psychoanalysis and psychopathology theories. Cohen and Özsoy (2021), citing Paulhus (2014), note that central to narcissism is dysfunction related to the management of an intense need for validation and admiration. Failure to effectively manage the above-mentioned needs ascribed to extreme or obstinate behavior or impaired regulatory capacities would result in negative psychological consequences, notably grandiosity or vulnerability (Wright et al., 2013). Pincus et al. (2009) and LeBreton et al. (2018) provide further insight into the profile of a narcissist in that the outward displays of grandiosity, self-serving and attention-seeking behaviors stem from the underlying suppressed feelings of jealousy, shame, low self-esteem, and emptiness.

Individuals with narcissistic tendencies are drawn to career roles that allow for the display of power, status and flattery (Peral, 2019) such as leadership roles. However, narcissists often overestimate internalized abilities to lead others successfully (Judge et al., 2006). Moreover, narcissists are known to take advantage of others and manipulate colleagues to obtain desired outcomes (LeBreton et al., 2018; Paulhus and Williams, 2002). In the workplace, employees displaying narcissistic traits can negatively influence the organizational environment and employee performance including but not limited to poor attendance, sharing of confidential information, or withholding effort (Grijalva and Newman, 2015). *Per se*, literature indicates that individuals exhibiting narcissistic traits create a toxic environment through destructive, manipulative, and attention-seeking behavior and interaction with colleagues and management (Aboramadan et al., 2020; Pincus et al., 2014). Known for self-centeredness and little to no display of empathy, narcissistic individuals often exhibit behavior toward others that can be perceived as aggressive; they can verbally or physically harass colleagues and engage in unethical behaviors, which can lead to a hostile working environment (Kurniawan et al., 2023).

Moreover, narcissism in a leadership context can positively and/or negatively influence workplace dynamics. Various research indicates that leaders with narcissistic traits are more likely to exhibit higher levels of confidence and charisma, which could influence and motivate their employees to perform (Gruda et al., 2022). On the contrary, those same traits can often come across as dominant, self-centeredness and a lack of empathy–resulting in decreased team morale and performance (Nevicka et al., 2018). Managers demonstrating narcissistic traits tend to put personal success and achievement above team- and organizational goals, which could result in critical decisions that might be deemed unethical (Xiao et al., 2018). This behavior not only creates a workplace culture that promotes manipulation and fear among employees, but it can also reduce the trust relationship between employees and management, whereby managers can exploit subordinates ascribed to seniority and entitlement (Gauglitz et al., 2022). Leaders displaying narcissistic traits might manage to foster short-term success of their team and subsequent performance; however, it can have undesirable long-term effects on the performance and well-being of the organization (Grijalva and Harms, 2014).

#### 3.1.2 Machiavellianism

The term Machiavellianism comes from literature by seminal authors, for example, Christie and Geis (1970). It is characterized by the ability to strategically manipulate others for personal gain, enjoyment and status (Dahling et al., 2009) in the absence of remorse and empathy coupled with a blatant disregard for following social norms and ethical codes of good practice (Christie and Geis, 1970; Dahling et al., 2009; Paulhus and Williams, 2002). Machiavellians will utilize calculated and manipulative tactics to get ahead (Peral, 2019) and likely seek roles that allow autonomy and freedom to perform job functions. Machiavellianism is positively correlated with aspects indicative of maladjustment, subsuming entitlement and being exploitative (Persson, 2019). Machiavellians focus on strategic manipulation and achieving power, often employing deceitful and unethical tactics without concern for others’ well-being (Jones and Paulhus, 2014).

Machiavellian leaders and managers often exhibit a high degree of strategic thinking and manipulative behavior, which are used to influence and control employees to achieve personal objectives (Wu et al., 2019). Individuals with Machiavellian characteristics tend to be highly self-interested and are willing to exploit others to maintain power and status (Back et al., 2013). In managerial roles, Machiavellians are adept at using flattery, deceit and manipulation to navigate organizational politics and ascend the corporate ladder (Kessler et al., 2010). Machiavellians prefer environments with significant autonomy and can operate without stringent oversight, allowing for the use of manipulative tactics more freely (Bereczkei et al., 2010).

The presence of Machiavellian managers can significantly impact on organizational culture and employee well-being (Gkorezis et al., 2015). The internalized focus on personal gain can lead to decisions that might not be in the organizations or their employees’ best interest, fostering a culture of fear and mistrust (D’Souza and de Lima, 2015). Employees under Machiavellian managers may experience higher stress levels and job dissatisfaction, often feeling exploited and unsupported (Maftei et al., 2022). This environment can increase turnover rates and decrease overall productivity (Padilla et al., 2015).

#### 3.1.3 Psychopathy

Psychopathy, considered the most malicious of the three Dark Triad traits, was first introduced into psychological discourse by Cleckley (1976) in his seminal work, titled *The Mask of Sanity*. Cleckley (1976) described psychopaths as superficially charming yet emotionally shallow and devoid of empathy and remorse. Based on Cleckley’s work, Hare (1991, 2003) developed the Psychopathy Checklist-Revised (PCL-R), which has become the benchmark for diagnosing psychopathy (LeBreton et al., 2018). The PCL-R operationalizes psychopathy through a range of traits, *inter alia*, ruthless manipulation, exploitative behavior, as well as the pursuit of power and success without regard for consequences (LeBreton et al., 2018). Psychopaths display no signs of remorse and lack empathy altogether by selfishly taking what they believe to be ‘owed’ or deemed rightfully theirs (O’Boyle et al., 2012). Moreover, psychopaths are known for being impulsive, irresponsible and cunning, with a predisposition toward counterproductive and criminal behavior (LeBreton et al., 2018; Williams et al., 2007). Individuals with psychopathic traits are impulsive (Spurk et al., 2015) and tend to lack remorse and empathy, gravitating toward jobs that involve significant risks but high rewards (Crysel et al., 2013).

Psychopathic leaders and managers are particularly detrimental to organizational and employee well-being (Smith and Lilienfeld, 2013). Impulsivity and lack of remorse often lead to careless decision-making, prioritizing personal gain over organizational success. These individuals are skilled at deceit and manipulation, allowing them to ascend to leadership positions where they can exert significant influence (Smith and Lilienfeld, 2013). In such roles, psychopathic managers might exploit employees, foster a toxic work environment, and engage in unethical practices without concern for repercussions (Boddy, 2016; Wu et al., 2019). The internalized tendency to view colleagues and subordinates as mere ‘means to an end’ or tools for achieving predetermined goals can result in widespread organizational dysfunction and high turnover rates (Mathieu et al., 2014). Boddy (2016) explains that the presence of psychopathic traits in leaders is deemed more damaging than Machiavellianism and narcissism, ascribed to the sheer lack of empathy and propensity for harmful behavior. Managers with psychopathic characteristics often lack the long-term strategic thinking seen in Machiavellians, instead opting for short-term gains achieved through unethical means (Barelds et al., 2018). This behavior undermines team trust and morale and puts the organization at risk for legal and reputational damage (Boddy, 2016).

### 3.2 Career interest

Hansen and Wiernik (2018) define career interests as the preference for a particular career or job role. Similarly, Rounds and Su (2014) expand on career interests by eluding that these preferences toward specific roles are based on individual traits, which motivate certain activities in accordance with the perception that positive outcomes are likely under such circumstances. Jonason et al. (2014) and Schneider et al. (2017) confirm that individuals tend to gravitate toward jobs or career roles that reflect internalized interests, personality, values, and environment. Understanding one’s vocational interests contributes to career-choice self- efficacy and well-informed career choices (Morgan and de Bruin, 2019). The propensity toward interest in specific career choices can be influenced by the underlying or perceived satisfaction from following a specific career path (Morgan et al., 2019). This notion is supported by Savickas (1995), who deems interests as a motivational construct from which an individual seeks to satisfy internalized wants and needs to achieve a sense of fulfilment and success (Rounds and Su, 2014). This approach is rooted in the social cognitive career development theory (SCCDT), which emphasizes the role of cognitive processes in shaping career-related behaviors and decisions.

The social cognitive career development theory (SCCDT), proposed by Lent et al. (1994), emphasizes the relationship between personal attributes, environmental factors, and behavior in shaping an individual’s career development (Adachi, 2004). According to the SCCD theory, career interests are influenced by self-efficacy beliefs, outcome expectations, and goals, which are, in turn, shaped by personality traits and past experiences (Levy and Myers, 2022).

Personality plays a crucial role in SCCDT as it impacts self-efficacy, which refers to an individual’s belief in his/her ability to perform tasks in specific domains (Appling et al., 2022). According to SCCDT, personality traits are antecedents that influence career interests as model outcomes moderated by self-efficacy beliefs. Other theories that should be taken into consideration, include, for example the Person-Job Fit Theory which could provide a nuanced understanding of how individuals with high Dark Triad traits navigate careers (Kristof-Brown et al., 2005). The Trait Activation Theory posits that certain environments trigger the behavioral expression of traits (Tett and Burnett, 2003). Holland’s theory of career choice, known as the RIASEC model, offers a more specific and widely used framework that directly links personality traits to occupational preferences (Holland, 1997). The RIASEC model categorizes individuals into six occupational types in accordance with dominant personality traits and career interests, *inter alia*, realistic, investigative, artistic, social, enterprising, and conventional. The RIASEC model is particularly relevant in understanding how individuals with certain personality traits, such as those associated with the Dark Triad traits, might struggle to find suitable career niches (Paleczek et al., 2018). Jonason et al. (2014) suggest that individuals exhibiting traits such as narcissism, Machiavellianism, or psychopathy often perform poorly in roles that do not align with their maladaptive personality characteristics, leading to dissatisfaction and increased engagement in counterproductive work behaviors (Paleczek et al., 2018). Consequently, understanding the specific career environments where these individuals might experience more favorable outcomes–while minimizing negative impacts on others and the organization–remains a crucial area for further research (Lyons, 2019).

### 3.3 Career success

Career success is invaluable for employees, as it is deemed a personal reflection of the sum of work, effort, time and energy invested in producing good work outcomes. Career success is not accomplished overnight and is often a long, stable journey, making the achievement rewarding. Heslin (2005b, p.376) poses the question: “*What is success defined as, what determines a successful career*?” The mentioned author puts forward a theory that career success culminates in multiple facets, *inter alia*, education, intellectual aptitude, personality traits, motivational drivers, status, ability to network and the potential to foster relationships through mentoring (Heslin, 2005b). Dries et al. (2008) opine that career success has evolved and has shifted from job outcomes to experiences indicative of one’s career journey.

Chu et al. (2015) further elaborates on the evaluation indicators of skilled employees’ career success, distinguishing between subjective and objective features. Subjective career success includes facets such as work-life balance, life satisfaction, and perception of success (Shockley et al., 2016), while objective success comprises tangible measures such as income level, wage growth, and promotion frequency (Heslin, 2005a). Objective career success has historically dominated the corpus of knowledge regarding defining and observing career success (Arthur and Rousseau, 1996; Gu and Su, 2016; Heslin, 2005a). Ng and Feldman (2014) define objective career success as the tangible, observable and measurable rewards an individual obtains for work performed, which could be verified by others. Researchers have since expanded on the mentioned definition by including that these observable rewards can be in the form of remuneration, promotions, and job status amongst others (Spurk et al., 2018). Career advancement, rewards, and recognition of service have also been mentioned (Shockley et al., 2016), while promotion history (Dries et al., 2008) and level of job title prestige (Briscoe et al., 2021) have additionally been included. Seibert et al. (2024) posit that, in the current changing world of work, employees are placing less emphasis on objective measures of rewards and success, primarily ascribed to these factors being influenced by external factors beyond the employee’s control.

Ascribed to objective success receiving less attention, subjective career success has garnered the necessary consideration (Ng and Feldman, 2014). Subjective career success is a multifaceted concept that involves an individual’s internal assessment of their career, emphasizing personal fulfilment, satisfaction, and intrinsic motivations rather than external rewards (Schultheiss et al., 2023). This notion encompasses intangible outcomes such as a sense of purpose, meaning, and the perceived impact of one’s work (Breland et al., 2007). Subjective career success incorporates perceptions of achievement, future perspectives, recognition, and career satisfaction (Dewi et al., 2022). Various factors influence subjective career success, including personal time, job security, social connections, challenging work assignments, organizational status, development opportunities, authenticity in the workplace, organizational commitment, considering one’s career as a calling, and overall well-being (Dai and Song, 2016; Shockley et al., 2016). Subjective career success reflects an individual’s subjective evaluations and emotional responses to career experiences (Yu et al., 2022), emphasizing internal perceptions and experiences gained throughout one’s career journey (Ng and Feldman, 2014). In essence, subjective career success represents a comprehensive evaluation of different aspects of one’s career, including emotional fulfilment, personal meaning, and the satisfaction derived from work experiences (Abele et al., 2010).

## 4 Development of propositions

### 4.1 Dark Triad traits and career interests

The relationship between career interests and the Dark Triad traits has been an area of growing research interest (Spurk et al., 2015). As conceptualized within the RIASEC framework, career interests reflect individuals’ preferences for specific work environments based on innate personality traits. According to Jonason et al. (2014), individuals scoring high on the Dark Triad traits tend to gravitate toward specific career niches that align with internalized personality characteristics.

Firstly, individuals with high levels of psychopathy might be inclined toward career niches that are practical, task-oriented, and involve minimal interpersonal interaction (Patrick and Drislane, 2014; Smith and Lilienfeld, 2013). The stated preference likely stems from discomfort with social norms and a tendency to prioritize personal gain over collaborative efforts (Neumann et al., 2012). Consequently, individuals scoring high in psychopathy might excel in isolated work environments where a lack of empathy and social connection has minimal impact on others (Anderson et al., 2018; Babiak and Hare, 2006). Specifically, participants scoring high on psychopathy would be drawn to realistic and practical careers. Similarly, research by Anderson et al. (2018) has indicated that individuals with a high level of psychopathy are more likely to pursue careers in which control can be exerted without being held accountable for social relationships. Notably, individuals with high levels of psychopathy favor realistic and investigative career environments, enabling the pursuit of independent tasks while maintaining a sense of authority. For example, research conducted by Mathieu et al. (2014) has shown that individuals in corporate environments scoring high in psychopathy frequently flourish in leadership positions, where others can be manipulated and outcomes influenced without regard for ethical standards. Moreover, Smith and Lilienfeld (2013) investigated how individuals scoring high in psychopathy typically thrive in high-stake, competitive settings where an individual advantage precedes teamwork.

Secondly, individuals scoring high in narcissism are often drawn to career paths that offer visibility, status and recognition, such as occupations in entertainment, politics, and management, where a desire for attention and leadership can be fulfilled (Liu et al., 2021). This preference for careers that provide opportunities for self-promotion and public recognition aligns with narcissists’ inclination toward roles where influence can be exerted over others and external validation for achievements is bestowed, making narcissists well-suited for positions involving leadership and public speaking (Martin et al., 2016). Being arguably the most social of the three traits, narcissists are self-centered and enjoy status and being admired by others (Jonason and McCain, 2012). Therefore, participants scoring high on narcissism are expected to perform well in artistic, social and enterprising niches (Jonason and McCain, 2012). While narcissists might excel in careers that cater to internalized career interests, the self- centered nature of narcissism can pose challenges in team-based settings where collaboration and humility are essential (Ding et al., 2023; Lyons, 2019). Individuals with higher levels of narcissism could find themselves drawn toward jobs involving positions of power and influence, where they can earn others’ praise and admiration, as discovered in a study conducted by Martin et al. (2016). Furthermore, Nevicka et al. (2018) found similar research outcomes; notably, individuals scoring higher in narcissism tended to pursue career roles involving public speaking and having the authority to make crucial decisions–in line with an internalized preference toward having power and influence over others. These results suggest that narcissism not only influences an individual’s career choices but also determines future job outcomes.

Moreover, narcissistic traits can have both positive and negative effects in the workplace. Studies have examined how a leader’s narcissism can influence employees’ career success through ingratiation and focusing on advancing their careers (Martin et al., 2016; Nevicka et al., 2018). Despite the potential benefits of narcissism in certain career domains, it is essential to consider how individuals with high levels of narcissism can manage interpersonal relationships and adapt to work environment demands that ensure sustained success (Gruda et al., 2022; Gupta and Misangyi, 2018). Understanding the interplay between narcissistic traits and career choices can provide valuable insights into the factors that drive individuals with narcissistic tendencies toward specific professions and how these traits influence their career trajectories (Davis and Brunell, 2012).

Lastly, individuals scoring high in Machiavellianism are often drawn to careers that allow them to exert control, influence others, and achieve personal gains, even at the expense of ethical considerations (Spurk et al., 2015). Enterprising careers involving leadership, persuasion, and negotiation are desirable to Machiavellians as these roles offer ample opportunities for manipulation and strategizing (Liyanagamage et al., 2022). Careers in business, politics, and law, where power dynamics and competition are prevalent, tend to appeal to individuals with Machiavellian traits (Liyanagamage et al., 2022). Machiavellians thrive in environments where organizational politics can be navigated, opportunities exploited, and positions of power can be obtained (Belschak et al., 2013). However, the tendency to prioritize personal gain over collaboration can result in ethical challenges and conflicts within teams (Xiu et al., 2023). Despite this, the ability to maintain a facade of charm and competence often assists Machiavellians to succeed in competitive and high-stakes environments, albeit sometimes at the expense of long-term team cohesion and organizational trust (O’Boyle et al., 2012). Machiavellians are recognized for their intelligence, adaptability, and resourcefulness, alongside negative, self-serving, and unethical personality traits (Shah et al., 2021). While Machiavellianism can lead to antisocial outcomes when paired with servant leadership behaviors, the adverse effects can be mitigated, enhancing perceived leadership effectiveness (Jonason et al., 2014).

Research conducted by Liyanagamage et al. (2022) established that individuals with higher Machiavellian traits are inclined to pursue careers that promote negotiation, leadership, and manipulation. Liyanagamage et al. (2022) posited that fields such as finance, law, and politics attract Machiavellians ascribed to the frequent rewards for those proficient at exploiting power dynamics and organizational politics. O’Boyle et al. (2012) corroborated this, finding that Machiavellians thrive in settings that enable them to maximize opportunities for self- advancement while preserving a facade of competence and integrity.

### 4.2 Dark Triad traits and career success

The Dark Triad traits have been associated with job performance and subjective success, highlighting the complex interplay between Dark Trait traits and career outcomes (O’Boyle et al., 2012; Furnham and Treglown, 2021). Considering the Dark Triad traits and career success, narcissists prefer to be the center of attention and would experience dissatisfaction and annoyance when working in teams requiring collaboration, resulting in conflict and decreased productivity (Lyons, 2019), adversely impacting career success. Recent research also corroborates the notion that narcissism can exert an additional influence on career success. Ding et al. (2023) assert that narcissists frequently seek ambitious careers that reflect internalized self-image. However, a deficiency in empathy and a propensity for self-promotion might generate workplace conflicts impeding sustained success (Ding et al., 2023). Narcissists excel in competitive environments, prioritizing individual achievement, but are less effective in collaborative contexts requiring teamwork (Gruda et al., 2022).

Furthermore, individuals high on Machiavellian traits might feel trapped, reacting in a disruptive and rebellious manner to break free when autonomy is restricted and stringent rules and regulations are imposed (Paleczek et al., 2018). Machiavellianism is associated with immediate career advancement in positions that value manipulation and strategic conduct. Research by Jonason et al. (2015) indicates that individuals exhibiting high levels of Machiavellianism tend to excel in competitive settings that permit deceptive and self-serving strategies. Nevertheless, these individuals frequently encounter enduring challenges, as overt manipulative behavior ultimately erodes organizational trust and cooperation (Spurk et al., 2015). Xiu et al. (2023) assert that Machiavellians might encounter difficulties in achieving career success when self-serving motives are revealed or when external conduct conflicts with organizational values, thereby diminishing prospects for enduring career advancement.

Finally, participants who score high on psychopathy with a mundane person-environment fit offering no risks or thrills, requiring a great deal of personal interaction, could become uninterested and, consequently, engage in irresponsible behavior and poor decision-making, which could result in adverse individual and organizational outcomes (O’Boyle et al., 2012) impeding work success (LeBreton et al., 2018). Whereas impulsiveness, apathy and unethical behavior might offer them short-term success, Smith and Lilienfeld (2013) found that these behaviors often lead to negative long-term consequences, such as job loss or a tarnished professional credibility. The mentioned outcomes are corroborated by research conducted by Mathieu et al. (2014), which found that individuals who engage in counterproductive work behaviors are more likely to hinder the ability to attain career success ascribed to counterproductive behavior, which often leads to team conflicts and distrust from co-workers.

### 4.3 Dark Triad traits as moderator between career interest and career success

Research indicates that the Dark Triad traits might impact emotional regulation and career planning, ultimately shaping individuals’ career trajectories (Ghosh and Sinha, 2024; Paleczek et al., 2018; Tariq et al., 2021). For example, narcissism can influence individuals’ career paths and career success in the workplace. Individuals with high levels of narcissism might be more likely to pursue careers that align with the desire for admiration and status (Back et al., 2013; Sanecka, 2021). However, if job roles do not provide the recognition envisioned, narcissists might engage in behaviors aimed at elevating perceived status at the expense of others (O’Boyle et al., 2012). This behavior can have a negative impact on job performance and, consequently, overall career success (Harms et al., 2022). The alignment or misalignment of individuals’ career interests with job roles can either enhance or diminish career outcomes, with narcissism playing a moderating role in this relationship (O’Boyle et al., 2012). Research by Grijalva and Harms (2014) found that narcissistic individuals show a preference toward careers with positions of influence, admiration and power. However, their lack of compassion for others and manipulative tactics might hinder their ability to maintain workplace relationships, resulting in dysfunctional team dynamics and negatively affecting their long- term success in environments requiring collaboration.

Machiavellianism, characterized by manipulativeness and strategic behavior, can significantly influence individuals’ career paths and success in the workplace (Cohen and Özsoy, 2021). Individuals high in Machiavellianism are likely to seek out careers where they can exert control and influence, leveraging manipulative strategies and willingness to engage in unethical behavior to gain a competitive advantage (Fatima et al., 2021). However, in environments where the ability to manipulate is restricted or where ethical standards are strictly enforced, Machiavellians might resort to unethical behavior to achieve predetermined goals, potentially harming long-term career success (Özsoy, 2018; Rizvi and Siddiqui, 2023). Research carried out by Spurk et al. (2018) discovered that although Machiavellianism correlates with immediate career benefits, the resultant decline in trust and interpersonal relationships might result in prolonged obstacles to career progression.

Individuals scoring high on psychopathy tend to gravitate toward careers that offer high-stake environments or isolated work settings where it is possible to operate with minimal oversight, aligning with the desire for autonomy and excitement (Babiak and Hare, 2006; Steinert et al., 2021). However, when placed in roles that do not provide the desired thrill or independence, individuals high in psychopathy might engage in reckless or irresponsible behavior, leading to poor job performance and diminished career success (Spurk et al., 2015). A recent study by Mathieu et al. (2014) suggests that individuals with psychopathic traits could initially succeed in career roles requiring risk-taking and competition; however, a disregard for workplace regulations and inclination to making unethical decisions frequently result in career instability and diminish career success. The impact of psychopathy as a moderator between career interest and career success is dependent upon the degree to which the career environment permits autonomy and risk-taking. When such opportunities are limited, psychopaths could display counterproductive work behaviors (Smith and Lilienfeld, 2013; Steinert et al., 2021), ultimately undermining career success.

## 5 Research method

### 5.1 Research design

A post-positivist research stance was adopted to investigate the nexus between the Dark Triad traits, career interests, and career success in that human behavior will be analyzed through objective reasoning (Yilmaz, 2013). The rationale for operationalizing a post-positivistic stance is that the researcher perceives reality as objective. Therefore, it can be measured by means of a quantitative survey (Bell et al., 2021). In accordance with the mentioned research paradigm, a quantitative research methodology was implemented, whereby numerical data was gathered and analyzed to predict the outcome of the latent variables (Yilmaz, 2013). The specific research design could be classified as cross-sectional and descriptive in nature. In a cross- sectional research design, data collection occurs at a single point in time (Leedy and Ormrod, 2013). The descriptive nature of the study aims to describe a phenomenon as accurately as possible in a specific target population (Salkind and Frey, 2021).

### 5.2 Population and sampling

The target population of the study was the economically active populace of South Africa. It is extrapolated that South Africa has an economically active population of approximately 16.7 million citizens as of the first quarter of 2024 (Statistics South Africa, 2024). A sample size of around 385 participants is considered sufficient on a 95% confidence level with a 5% margin of error, ensuring reliable results that can be generalized to the target population (Adam, 2020). Ascribed to the research under discussion being conducted within the South African workplace context, the inclusion criteria used to draw the sample were age, work experience and English language proficiency. More specifically, eligible participants had to be 18 years or older and have at least two or more years’ working experience. Within the South African context secondary schooling is compulsory until Grade 12 which would equate to 18 years of age. Thus, only employed of-age participants were eligible for inclusion in the study. English language proficiency is deemed necessary since the assumption would be that the sample comprehends the questionnaire items and could mindfully complete the measuring instrument (Mabazo and Van der Walt, 2024). A non- probability sampling technique was used to generate the sample, viz., participants do not have an equal chance to participate in the study (Rahman, 2023). More specifically, purposive convenience sampling was operationalized, which implies that participants are readily available and willing to participate (Scholtz, 2021). Convenience sampling is a commonly used method in research that allows researchers to gather data without the logistical challenges associated with random sampling (Jager et al., 2017).

### 5.3 Research participants

The final sample included *N* = 406 economically active participants. The sample was almost equally divided in terms of gender, with 52.6% (*n* = 211) female and 47.4% (*n* = 190) male participants. Considering the age distribution, the average age was 37 years and 2 months, with a STD of 10.602. Regarding ethnicity, 54.9% (*n* = 220) of the participants were African, 37.9% (*n* = 152) were white, 4.5% (*n* = 18) colored and 2.7%, representing 11 participants, were Indian. Pursuantly, 46.9% (*n* = 188) of the sample spoke an indigenous language at home, followed by Afrikaans (*n* = 120; 29.9%) and English (*n* = 93; 23.2%). Considering the highest academic qualification, most of the sample held a certificate or diploma (*n* = 125; 31.2%), followed by a grade 12 (*n* = 96; 23.9%), a bachelor’s qualification (*n* = 82; 20.4%), an honors degree (*n* = 53; 13.2%) and a magister degree (*n* = 9; 2.2%). Considering the employment typology, the vast majority were full-time employed (*n* = 364; 90.8%), followed by participants working part-time on a contract (*n* = 19; 4.7%) and 3% representing 12 sole proprietors. Lastly, six participants, representing 1.5% of the sample, freelanced. Regarding employee rank, most of the participants were employees (*n* = 153; 38.4%), followed sequentially by middle management (*n* = 111; 27.9%), senior or top management (*n* = 73; 18.3%), supervisor or line manager (*n* = 49; 12.3%) and sole proprietor (*n* = 12; 3%). Lastly, when considering the work experience of participants, most of the sample had work experience.

### 5.4 Measuring instruments

Primary data was collected using an electronic structured measuring instrument consisting of four sections. Section A comprised self-reporting demographic items used to provide a sample profile. It should be noted that demographic information has not been used in any statistical analysis. Questionnaire items required details underscoring the participant’s age, gender, race, language, and education. Section B consisted of the SACII-Short which contains 30 items utilizing an emoji response format ascribed to the inherent affective association while rating career interests (Morgan, 2022). The SACII-short has been shown to have improved reliability and fit with the overall circumplex structure of the RIASEC scale due to the emotional response required when answering items regarding individuals’ career interests (Phan et al., 2017; Naidu, 2020). It is assumed that when facial expressions in the form of an emoji are used in conjunction with words or feelings, participants can better rate likes or dislikes toward an item (Evans, 2015; Kılıç et al., 2021). The SACII- Short response format, therefore, consists of emojis representative of the corresponding response categories, viz. strongly dislike, dislike, unsure, like and strongly like (Morgan, 2022). An example statement is, *‘Do routine maintenance of machines*.’ The six sub-scales, *inter alia,* realistic, investigative, artistic, social, enterprising and conventional, have model-fit reliability coefficients ranging from 0.72 to 0.83 (Morgan and de Bruin, 2019). Therefore, the SACII-Short demonstrates satisfactory psychometric properties for use within the South African context (Morgan, 2022).

Section C measured the Dark Triad traits using the Dark Triad Dirty Dozen (DTDD). The previously mentioned questionnaire, developed by Jonason and Webster (2010), is a short and concise measure of the Dark Triad traits, comprising three sub-scales underscoring narcissism, Machiavellianism and psychopathy. The DTDD aims to create a single measure for the three traits, which were previously assessed separately using 91 scale items, which could be tedious and time-consuming for participants, potentially leading to response fatigue (Jonason and Webster, 2010). The DTDD consists of three sub-categories comprising four items, each requiring participants to rate statements on a five-point Likert-type scale ranging from 1 (strongly disagree) to 5 (strongly agree). Examples of the items subsume, ‘*I tend to manipulate others to get my way*’ (Machiavellianism), ‘*I tend to be cynical’* (Psychopathy) and ‘*I tend to seek prestige or status*’ (Narcissism). Satisfactory reliability was reported for the DTDD with Cronbach’s alpha coefficients ranging from 0.78 for Machiavellianism, *α* = 0.80 for psychopathy and α = 0.83 for narcissism. The overall item reliability coefficient for the DTDD was 0.82 (Jonason and Webster, 2010).

Section D measured subjective career success utilizing the subjective career success inventory. The subjective career success inventory (SCSI), developed by Shockley et al. (2016), consists of 24 items, measuring items relating to recognition, quality work, meaningful work, influence, authenticity, personal life, growth and development as well as satisfaction which combined constitute subjective career success. Responses were indicated on a five-point Likert-type scale, with response categories ranging from ‘not at all (1) to ‘a great deal’ (5). Questions posed included, for example, *‘My supervisor has told me I do a good job* (recognition)*,’; ‘I am proud of the quality of the work I have produced’* (quality work) and *‘I believe my work has made a difference’* (meaningful work). Shockley et al. (2016) reported high alpha values for the overall SCSI (α = 0.94) and for specific dimensions, ranging from 0.77 to 0.92.

### 5.5 Statistical analysis

Statistical analyses were conducted using the Statistical Package for Social Sciences (IBM SPSS) version 29, SPSS AMOS version 29, and SPSS Process Macro version 4.3 (model 4). Data screening procedures were implemented to ensure data consistency and accuracy, including testing assumptions of normality and linearity (O’leary, 2021). Normality can be assessed by obtaining skewness and kurtosis values (Pallant, 2011) with cut-off values smaller than ± 2 for skewness and ± 4 for kurtosis indicative of a normal distribution (Mabitsela et al., 2024). Descriptive statistics (viz. mean, median standard deviation) were calculated to summarize the data distribution (Zake et al., 2024a). To ensure the reliability of the measurement scales, Cronbach’s alpha coefficients were computed for each construct, and values above 0.70 were considered acceptable (Tavakol and Dennick, 2011). Composite reliability was assessed, values exceeded the recommended threshold of 0.7 (Zake et al., 2024b). Construct validity was assessed through exploratory factor analysis (EFA) and confirmatory factor analysis (CFA) (Ghiyasvandian et al., 2017). Discriminant validity was confirmed by ensuring that each construct’s average variance extracted (AVE) exceeded the maximum shared variance (MSV) as per Zake et al. (2024b). An unrotated EFA was used to control for common method bias by means of determining whether a single factor explains more than 50% of the variance (Aguirre-Urreta and Hu, 2019). Structural equation modeling (SEM) in SPSS Amos was performed to estimate the theoretical model and calculate the model fitness indices (Ramlall, 2016). SEM is a statistical technique to test complex relationships among latent variables (Hancock et al., 2019). Key indices for evaluating model fit include the chi-square statistic, comparative fit index (CFI), Tucker-Lewis index (TLI), and root mean square error of approximation (RMSEA) (Hancock et al., 2019). Generally, acceptable fit thresholds subsume CFI ≥ 0.95, TLI ≥ 0.95, and RMSEA < 0.08 (Scholtz et al., 2024).

Bivariate associations between variables were assessed using Pearson’s product–moment correlation, providing insights into the strength and direction of the correlation between measured constructs (Headrick, 2016). The following criteria were used to interpret the results, namely *r* = 0.1 to *r* = 0.29 (small effect), *r* = 0.30 to *r* = 0.49 (medium effect) and *r* = 0.50 to *r* = 1.0 (large effect) (see Botha et al., 2023). The bias-corrected percentile bootstrap method was performed to ascertain the moderating effect of the Dark Triad traits, with 95% lower-level (LLCI) and upper-level (ULCI) ranges excluding zero (McCallaghan et al., 2019). SPSS Process Macro version 4.3 was utilized to calculate the moderation analysis (Field, 2024).

### 5.6 Ethical considerations

Ethics clearance to conduct the research study was obtained from the University of Johannesburg’s Industrial Psychology and People Management Ethics Committee (reference number IPPM-2024-875 M). The standard ethical protocol was observed, namely informed consent, voluntary participation, confidentiality, anonymity and benevolence (i.e., no psychological or physical harm). Ascribed to the psychological nature of the Dark Triad traits, which might cause psychological discomfiture for participants, the contact details for the South African Depression and Anxiety Group (SADAG) were provided.

## 6 Results

### 6.1 Assessment of measuring instrument and model

The psychometric properties of the measuring instrument, including reliability and validity, were evaluated with results displayed in Table 1.

**Table 1:** Psychometric properties of the measuring instrument.

Scale	Cronbach’s alpha	Construct validity		
		CR	AVE	MSV
Career interest	0.896	0.972	0.536	0.201
Realistic career interest sub-scale	0.901			
Investigative career interest sub-scale	0.815			
Artistic career interest sub-scale	0.836			
Social career interest sub-scale	0.795			
Enterprising career interest sub-scale	0.782			
Conventional career interest sub-scale	0.855			
Dark Triad traits	0.865	0.938	0.557	0.306
Narcissism sub-scale	0.785			
Psychopathy sub-scale	0.812			
Machiavellianism sub-scale	0.877			
Subjective career success	0.938	0.963	0.513	0.372

CR, composite reliability; AVE, average variance extracted; MSV, maximum shared variance.

According to Table 1, career interest had a Cronbach’s alpha coefficient of 0.896 which can be deemed acceptable. Cronbach’s alpha coefficients for the career interest sub-scales ranged between α = 0.782 and α = 0.901. The Dark Triad traits had a Cronbach alpha coefficient of α = 0.865 (acceptable), while the alpha coefficients for the sub-scales ranged between α = 0.785 and α = 0.877. Lastly, the Cronbach’s alpha of subjective career success was 0.938, which is excellent. The composite reliability scores exceeded the recommended threshold of 0.7, whereas the convergent validity AVE scores were 0.5 and above (Cheung et al., 2024). Specifically, the scores were as follows: career interest (CR = 0.972; AVE = 0.536), Dark Triad traits (CR = 0.938; AVE = 0.557) and subjective career success (CR = 0.963; AVE = 0.513). Based on these results, the convergent validity of constructs in the measurement model was supported. Discriminant validity was ascertained, indicative of the constructs varying significantly from each other, as seen from the AVE scores exceeding the MSV values (Zake et al., 2024b). Based on the preceding results, the measuring instruments are considered reliable and valid.

### 6.2 Common method bias

Common method bias occurs when both dependent and independent variables are measured using a Likert-type scale, resulting in correlation parameter estimate bias (Du Plessis, 2023) which might be the case in the research reported on. Controlling for common method bias entails computing an exploratory factor analysis (EFA) with an unrotated factor solution to ascertain the number of factors that account for 50% of the variance (Du Plessis, 2023). Preliminary results indicate that the Kaiser-Meyer-Olkin (KMO) test for sampling adequacy was 0.863, and Bartlett’s test of sphericity had a statistically significant *p*-value on the 99th percentile (χ2 = 15886.422; df = 2,145; *p* = 0.000**). An unrotated factor analysis was performed, which indicated that six factors accounted for 49.136% of the variance. Pursuantly, factor 1 only accounted for 18.141% of the variance. Based on these results, common method bias did not occur in the research reported on.

### 6.3 Factor analysis

Considering the factor structure of the different sub-scales of the questionnaire, exploratory factor analysis (EFA) with oblique rotation was carried out for the SCSI. Preliminary tests for the SCSI reverted a KMO-value of 0.915, and Bartlett’s test of sphericity was statistically significant (χ2 = 6497.113; df = 276; *p* = 0.000**). Results indicated that five components had an eigenvalue exceeding 1, accounting for 70.03% of the variance regarding subjective career success. The factor loadings ranged from 0.873 to 0.435.

Similarly, an EFA with oblique rotation was performed for the SACII-Short measuring career interests. The KMO-value was 0.863, and Bartlett’s test of sphericity was statistically significant (χ2 = 5953.558; df = 435; *p* = 0.000**). Furthermore, six components had eigenvalues exceeding 1, accounting for 63.252% of the variance in career interest. Factor 1 reverted factor loadings ranging between 0.825 and 0.657. Factor 2 reverted factor loadings ranging from 0,820 to 0.841. The factor loadings for factor 3 ranged between 0.833 and 0.679, while the factor loadings for factor 4 ranged between 0.822 and 0.445. Lastly, factor loadings for factor 5 ranged between 0.785 and 0.519, while the same for factor 6 ranged between 0.783 and 0.609. The factor analysis results were mostly in accordance with previous research (Morgan and de Bruin, 2019).

Considering the last scale, DTDD, measuring the dark triad traits, the KMO-value was 0.860, and Bartlett’s test of sphericity was statistically significant (χ2 = 2070.844; df = 66; *p* = 0.000**). Moreover, three components had eigenvalues exceeding 1, accounting for 65.585% of the variance. Factor 1 accounted for 40.916% of the variance, underscoring psychopathy, with factor loadings ranging from 0.519 to 0.807. Factor 2 accounted for 14.095% of the variance and underscored narcissism, while factor 3 accounted for 10.573% of the variance underscoring Machiavellianism. The factor analysis results were mostly in accordance with previous research. Also, factor loadings were mostly above 0.50 (Scholtz et al., 2024). High factor loadings suggest that the factor explains a significant portion of the variance in the observed variables, which is essential for the validity of the model (Beauducel and Frank, 2014).

### 6.4 Measure model fitness

To ascertain whether the structural model is appropriate for the data and, therefore, suitable for further analysis, structural equation modeling (SEM) with a maximum likelihood estimation in SPSS Amos 28 was calculated. Results reverted a minimum fit (chi-square/degree of freedom [CMIN/df] = 158.428; normed fit index [NFI] = 1.00; Tucker-Lewis index [TLI] = 1.00; comparative fit index [CFI] = 1.00; root mean square error of approximation [RMSEA] = 0.623). Goodness fit evaluates the overall performance of the measurement model; nevertheless, there is no threshold that allows for determining statistical significance (Mokoena et al., 2022). However, acceptable fit thresholds, *inter alia*, CFI ≥ 0.95 and TLI ≥ 0.95 were achieved (Scholtz et al., 2024). Therefore, the structural model was deemed fit and appropriate for supplementary analysis.

### 6.5 Descriptive analysis results

Table 2 provides an indication of the descriptive results computed for the latent variables. In terms of a realistic career interest the mean score was 12.62 (SD = 5.501) and the median was.

**Table 2:** Descriptive results and normality indicators.

Variable	Min	Max	Mean	Median	SD	Skewness	Kurtosis
CI	37.00	150.00	97.74	98.00	19.15	−0.101	0.241
RCI	5.00	25.00	12.62	12.00	5.501	0.199	−1.008
ICI	5.00	25.00	15.13	16.00	5.146	−0.234	−0.625
ACI	5.00	25.00	17.15	18.00	5.141	−0.451	−0.397
SCI	5.00	25.00	18.63	19.00	4.469	−0.617	−0.096
ECI	5.00	25.00	19.09	19.00	3.993	−0.528	−0.126
CCI	5.00	25.00	15.12	15.00	5.461	0.012	−0.825
Nar	4.00	20.00	9.27	9.00	4.05	0.042	−0.610
PP	4.00	17.00	6.62	6.00	6.70	1.066	0.845
Mac	4.00	20.00	8.24	8.00	3.49	0.579	−0.322
CS	44.00	120.00	95.73	97.00	14.46	−0.520	0.048

CI, career interest; RCI, realistic career interest; ICI, investigative career interest; ACI, artistic career interest; SCI, social career interest; ECI, enterprising career interest; CCI, conventional career interest; Mac, Machiavellianism; PP, psychopathy; Nar, narcissism; CS, career success.

12.00. Thus, participants endorsed response categories reflecting a positive inclination or liking in support of a realistic career interest. On the other hand, the mean score for an investigative career interest was 15.13 (SD = 5.146) and the median was 16.00. Hence, participants selected response options indicating a dislike in investigative career interests. Similarly, the mean score for an artistic career interest was 17.15 (SD = 5.141) and the median was 18.00. Therefore, participants selected response options indicating a dislike in artistic career interests. Also, the mean score for a social career interest was 18.66 (SD = 4.469) and the median was 19.00. Therefore, participants selected response options indicating a dislike in social career interests. In contrast, the mean score for enterprising career interests was 19.09 (SD = 3.993) and the median was 19.00 indicative of participants endorsing response categories reflecting a positive inclination or liking in support of an enterprising career interest. Lastly, considering a conventional career interest participants endorsed response categories reflecting a positive inclination or liking in support of a conventional career interest as evident from a mean score of 15.12 (SD = 5.461) and a median score of 15.00. In terms of subjective career success, the mean score for the sample was 95.73 (SD = 14.46), while the median was 97.00. Thus, considering career success the lower mean scores indicated that participants were experiencing less career success. On the other hand, the mean scores for the Dark Triad traits were above the median. Therefore, results were positively skewed, with participants endorsing response options that reflect higher levels of Dark Triad traits, for example, manipulating others, using deceit or flattery, and exploiting others, to mention a few. Skewness and kurtosis values were within the set threshold values indicating a normal distribution (Mabitsela et al., 2024).

### 6.6 Correlation analysis

Since the assumption of univariate normality was met, Pearson product–moment correlation was computed to ascertain the strength and direction of the association between the latent variables, with results displayed in Table 3.

**Table 3:** Bivariate correlation matrix.

Var	RCI	ICI	ACI	SCI	ECI	CCI	NAR	PP	MaC	CS
RCI	1									
ICI	0.423^**^	1								
ACI	0.138^**^	0.395^**^	1							
SCI	0.095	0.217^**^	0.461^**^	1						
ECI	0.196^**^	0.208^**^	0.329^**^	0.450^**^	1					
CCR	0.326^**^	0.224^**^	0.218^**^	0.360^**^	0.472^**^	1				
NAR	−0.064	−0.005	0.051	−0.036	−0.012	−0.048	1			
PP	0.095	−0.050	−0.116^*^	−0.173^**^	−0.111^*^	−0.105^*^	0.434^**^	1		
MaC	0.032	0.001	0.032	−0.105^*^	0.030	−0.068	0.454^**^	0.494^**^	1	
CS	0.080	−0.049	−0.004	0.224^**^	0.350^**^	0.202^**^	−0.119^*^	−0.211^**^	−0.194^**^	1

Var, variables; RCI, realistic career interest; ICI, investigative career interest; ACI, artistic career interest; SCI, social career interest; ECI, enterprising career interest; CCI, conventional career interest; Mac, Machiavellianism; PP, psychopathy; Nar, narcissism; CS, career success. *p ≤ 0.05; **p ≤ 0.01.

As per Table 3, the various facets of the SACII-Short questionnaire, viz. realistic, investigative, artistic, social, enterprising and conventional career interests statistically significantly correlated with each other on the 99th percentile except for social and realistic career interests. Moreover, the correlation between narcissism and the various career interest facets did not yield statistically significant results. Furthermore, psychopathy had small statistically significant correlations with artistic career interest (*r* = −0.116; *p* ≤ 0.05), social career interest (*r* = −0.173; *p* ≤ 0.01), enterprising career interest (*r* = −0.111; p ≤ 0.05) and conventional career interest (*r* = − 0.105; *p* ≤ 0.05). The correlations were negative, indicative of an inverse relationship. *Per se*, there would be a decrease in the career interest facets mentioned with an increase in psychopathy. On the other hand, psychopathy had a medium statistically significant correlation with narcissism on the 99th percentile (*r* = 0.434; *p* ≤ 0.01). The relationship was positive, and therefore, as psychopathy increases there is a concomitant increase in narcissism. Machiavellianism had a small statistically significant negative correlation with social career interest (*r* = −0.105; *p* ≤ 0.05). While Machiavellianism had medium statistically significant positive correlations with both narcissism (*r* = 0.454; *p* ≤ 0.01) and psychopathy (*r* = 0.494; *p* ≤ 0.01). Lastly, subjective career success had small statistically significant positive correlations with social (*r* = 0.224; *p* ≤ 0.01) and conventional career interests (*r* = 0.205; *p* ≤ 0.01) on the 99th percentile. Although, subjective career success had a medium statistically significant correlation with enterprising career interest (*r* = 0.350; *p* ≤ 0.01). On the contrary, subjective career success exhibited small negative associations with the Dark Triad traits, specifically Machiavellianism (*r* = −0.194; *p* ≤ 0.01), psychopathy (*r* = −0.211; *p* ≤ 0.01) and narcissism (*r* = −0.119; *p* ≤ 0.05).

**Figure 1:**
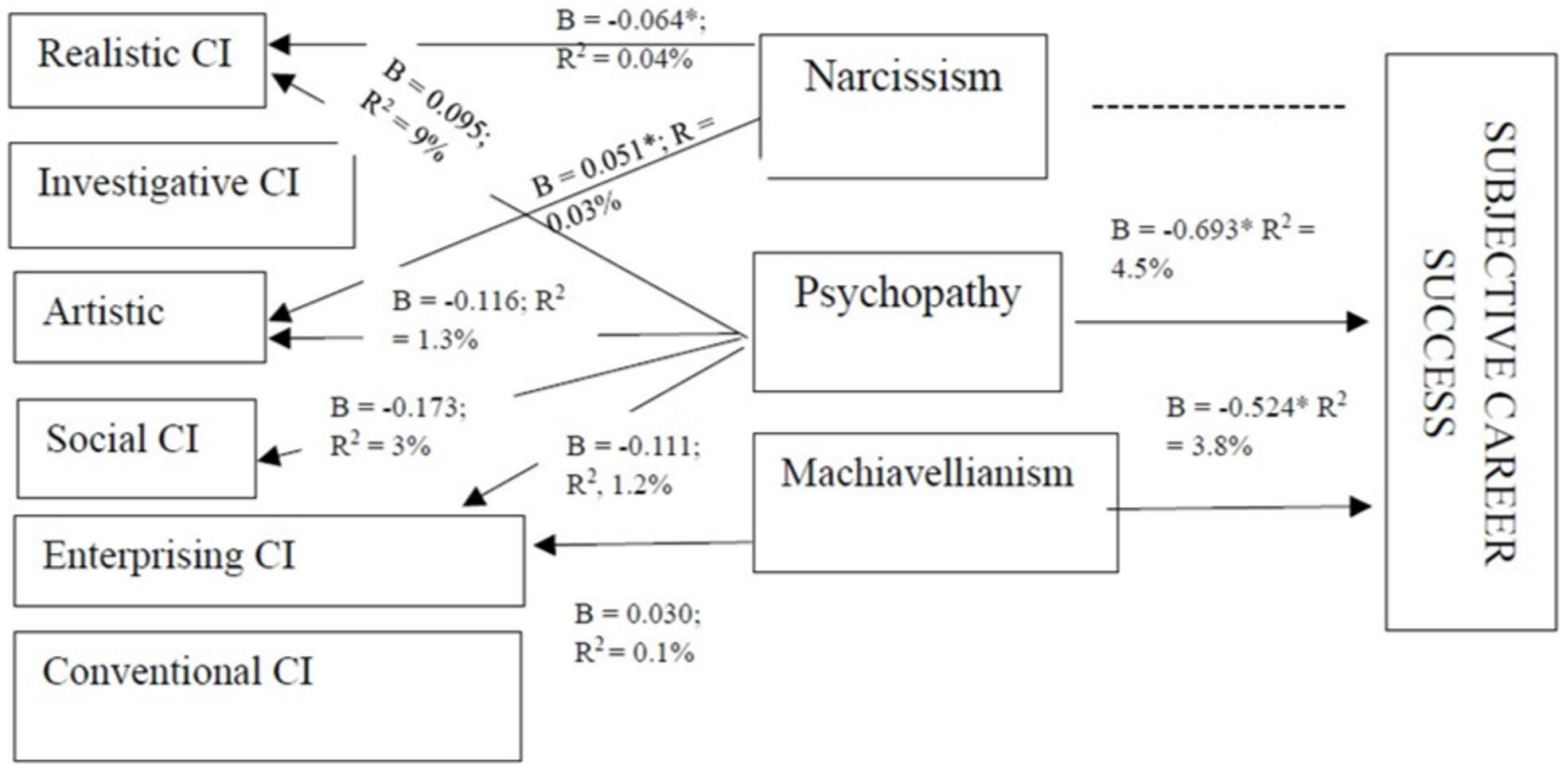
Significant direct path results for the SEM analysis CI, Career Interest; β, standardized beta coefficient; *R*^2^, R-square thus proportion variance explained. *, Does not include zero.

### 6.7 Structural equation modeling

To provide statistical evidence for the direct effects, standardized path results are provided in Table 4 and significant results are graphically depicted in Figure 1.

**Table 4:** Path results for the structural model.

Ho	Path	*β*	S.E.	C.R.	*p*
H1a	Realistic career interest ← narcissism	−0.185	0.067	−2.739	0.006**
H1b	Investigative career interest ← narcissism	0.014	0.064	0.225	0.822
H1c	Artistic career interest ← narcissism	0.126	0.063	2.009	0.045*
H1d	Social career interest ← narcissism	0.067	0.054	1.233	0.218
H1e	Enterprising career interest ← narcissism	0.013	0.049	0.259	0.795
H1f	Conventional career interest ← narcissism	0.006	0.067	0.083	0.934
H1g	Realistic career interest ← Psychopathy	0.291	0.101	2.570	0.004**
H1h	Investigative career interest ← Psychopathy	−0.133	0.096	−1.393	0.164
H1i	Artistic career interest ← Psychopathy	−0.386	0.094	−4.094	0.000**
H1j	Social career interest ← Psychopathy	−0.293	0.082	−3.584	0.000**
H1k	Enterprising career interest ← Psychopathy	−0.252	0.074	−3.432	0.000**
H1l	Conventional career interest ← Psychopathy	−0.195	0.101	−1.928	0.054

*p ≤ 0.05; **p ≤ 0.01.

**Figure 2:**
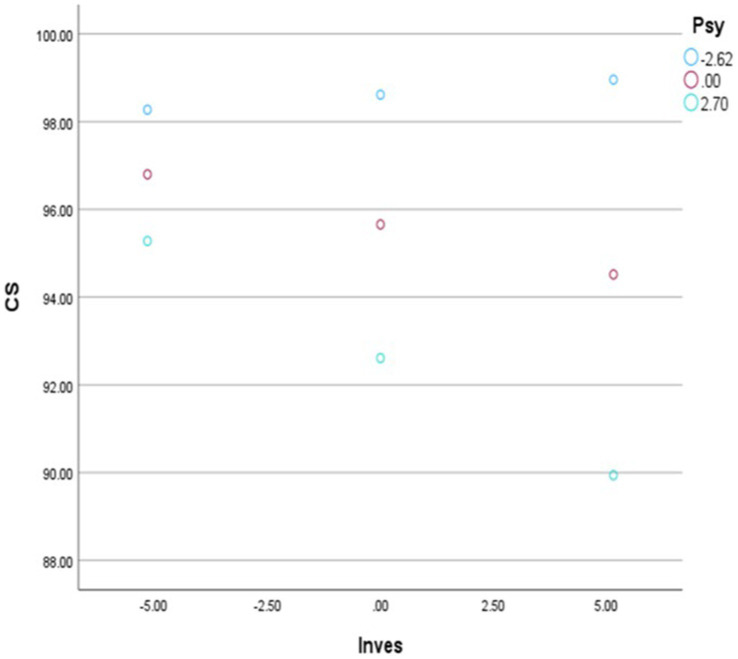
Slope analysis with psychopathy as moderator.

The structural paths tabulated in Table 4 indicated that narcissism had a negative effect on realistic career interests (*β* = 0.185; SE = 0.067; *p* = 0.006**) and a positive effect on artistic career interests (*β* = 0.126; SE = 0.063: *p* = 0.045*), providing support for hypotheses 1a and 1c. More specifically, 0.4% of the variance in realistic career interest and 0.3% of the variance in artistic career interest could be attributed to narcissism (see Figure 1). Furthermore, psychopathy had a positive effect on realistic career interest (*β* = 0.291; SE = 0.101; *p* = 0.004**). On the other hand, psychopathy had negative effects on artistic (*β* = −0.386; SE = 0.094; *p* = 0.000**), social (*β* = −0.293; SE = 0.082; *p* = 0.000**) and enterprising career interests (*β* = 0.252; SE = 0.074; *p* = 0.000**). *Per se*, 9% of the variance in realistic career interest could be attributed to psychopathy, while 1.3% of the variance in artistic, 3% of the variance in social and 1.2% of the variance in enterprising career interests could be attributed to the same. Based on the results presented hypothesis 1 g, hypothesis 1i, hypothesis 1j and hypothesis 1 k were accepted. Additionally, Machiavellianism reverted a positive effect on enterprising career interest (*β* = 0.124; SE = 0.057; *p* = 0.029*). As seen in Figure 2, only 0.1% of the variance in enterprising career interest could be attributed to Machiavellianism. As a result, hypothesis 1q was accepted.

Furthermore, psychopathy had a negative influence on subjective career success (*β* = −0.693; SE = 0.260; *p* = 0.008). Specifically, 4.5% of the variance in subjective career success could be attributed to psychopathy. Therefore, hypothesis 2b was accepted. Lastly, Machiavellianism had a negative effect on subjective career success (*β* = −0.524; SE = 0.199; *p* = 0,008). *Per se*, 3.8% of the variance in subjective career success could be attributed to Machiavellianism. Consequently, hypothesis 2c was accepted.

### 6.8 Moderation analysis

Moderation models were computed by means of PROCESS version 4.3 macro in SPSS (model 4) developed by Hayes (2018) (see, for example, Field, 2024). Subjective career success was the outcome variable, with career interest facets as predictors. The Dark Triad traits were the moderators, inter alia, narcissism, psychopathy and Machiavellianism.

The results of the moderation analyses were summarized in Table 5, indicating that the interaction between investigative career interest and psychopathy was statistically significant (*β* = 0.0539; *t* = −2.0431; *p* = 0.042*; 95%; CI [−0.2163 to −0.0042]). This indicates that psychopathy negatively moderates the relationship between investigative career interest and career success. Where high levels of psychopathy deteriorate the relationship between investigative career interest and career success. In terms of the model summary, the first regression between investigative career interest and psychopathy explained 5.8% of the variance in career success (*R*2 = 0.0580; *F* = 8.0901; *p* = 0.000). The second model, which added the interaction term (investigative career interest x psychopathy) explained an additional 1% of the variance (Δ*R*2 = 0.0100; *F* = 4.1741; *p* = 0.0417). The total variance explained was 6.8%. In accordance with the results presented, hypothesis 3 h was accepted. The other interactions were statistically insignificant, and the hypotheses related thereto were rejected. Furthermore, the Johnson-Neyman significance range indicates that at the point where psychopathy exceeds 0.6982, the moderation effect is statistically significant (*p* < 0.05). Once above this value, there is no significant influence of investigative career interest on career success.

**Table 5:** Moderation analysis results.

H0	Interaction	Coeff	SE	*t*	*p*	LLCI	ULCI
Narcissism as moderator							
H3a	RCI × Nar	0.0143	0.0325	0.4390	0.6609	−0.0497	0.0782
H3b	ICI × Nar	0.0284	0.0333	0.8526	0.3944	−0.0371	0.0940
H3c	ACI × Nar	−0.0043	0.0336	−0.1286	0.8977	−0.0703	0.0617
H3d	SCI × Nar	−0.0181	0.0378	−0.4795	0.6318	−0.0924	0.0562
H3e	ECI × Nar	−0.0233	0.0410	−0.5669	0.5711	−0.1039	0.0574
H3f	CCI × Nar	0.0286	0.0300	0.9531	0.3411	−0.0304	0.0875
Psychopathy as moderator							
H3g	RCI × PP	−0.0184	0.0485	−0.3789	0.7050	−0.1137	0.0770
H3h	ICI × PP	−0.1102	0.0539	−2.0431	0.0417*	−0.02163	−0.0042
H3i	ACI × PP	−0.9538	0.0500	−1.0763	0.2825	−0.1520	0.0444
H3j	SCI × PP	−0.0125	0.0559	−0.2228	0.8238	−0.1223	0.0974
H3k	ECI × PP	0.0084	0.0715	0.1181	0.9061	−0.1322	0.1491
H3l	CCI × PP	−0.0650	0.0503	−1.2913	0.1974	−0.1638	0.0339
Machiavellianism as moderator							
H3m	RCI × Mac	−0.0020	0.0379	−0.0520	0.9585	−0.0764	0.0725
H3n	ICI × Mac	−0.0116	0.0402	−0.2895	0.7723	−0.0907	0.0674
H3o	ACI × Mac	−0.0090	0.0377	−0.2376	0.8123	−0.0832	0.0652
H3p	SCI × Mac	−0.0671	0.0438	−1.5314	0.1265	−0.1531	0.0190
H3q	ECI × Mac	−0.0247	0.0488	−0.5055	0.6135	−0.1205	0.0712
H3r	CCI × Mac	−0.0086	0.0362	−0.2368	0.8129	−0.0797	0.0625

RCI, realistic career interest; ICI, investigative career interest; ACI, artistic career interest; SCI, social career interest; ECI, enterprising career interest, CCI, conventional career interest; CS, career success; L 95% CI, Lower 95% confidence interval; U 95% CI, Upper 95% confidence interval. *, Does not include zero.

As can be seen from Figure 2, the slope analysis represents the moderation effect of psychopathy on the relationship between investigative career interest and career success. The slope shows that as psychopathy increases, the relationship between investigative career interest and career success deteriorates. This indicates that the higher levels of psychopathy an individual has, the lower their career success when pursuing investigative career fields. When psychopathy is high (2.270) investigative career interest has little effect on career success. However, at low levels of psychopathy (−2.62), the influence of investigative career interest on career success increases significantly. Figure 2 supports the moderation hypothesis underscoring the important role of psychopathy in minimizing the contribution of investigative career interest on the experience of subjective career success.

## 7 Discussion

Psychopathy exhibited a statistically significant adverse effect on artistic, social and enterprising career interest, which can be explained by the disruptive characteristics of psychopathic traits. Individuals presenting with high levels of psychopathy could possibly have career interests consistent with their personality traits, such as competitive or risk-taking environments (Steinert et al., 2021). The adverse impact on career interests may suggest that these individuals find it challenging to pursue their interests consistently. This corresponds with existing research indicating that psychopathy is defined by impulsiveness, apathy and antisocial behaviors (Miller et al., 2017), all of which can hinder an individual’s capacity to remain committed to chosen career pursuits long term. This finding is also consistent with research that demonstrates that individuals presenting with psychopathic traits can be detrimental within the organizational context, where they might have trouble concentrating on long-term career objectives or sustaining a consistent level of engagement to job role ascribed to manipulative and unpredictable behavior (LeBreton et al., 2018). In essence, psychopathic traits impede the natural progression from career interests to career success due to interpersonal and motivational challenges. On the other hand, psychopathy reverted a positive influence on realistic career interests. As such, Kowalski et al. (2017) reported that psychopathy correlated positively with science, biology and business career interests. While a negative correlation was reported with social and work style factors (Kowalski et al., 2017). The reported findings support previous research findings. In line with existing literature, the negative effect of psychopathy on subjective career success could undermine longitudinal vocation success, as evidenced by psychopathic traits such as superficial charm, impulsiveness and lack of remorse (Boddy, 2016). The research results presented indicated that 4.5% of the variance in subjective career success could be attributed to psychopathy. Therefore, even though individuals presenting with psychopathic traits find positions that match innate career interests, such individuals might struggle to achieve career success in roles that require consistent effort, collaboration with others and establishing trust (LeBreton et al., 2018; Harms et al., 2022).

Furthermore, narcissism exhibited a statistically significant adverse effect on realistic career interests, and a positive effect on artistic career interests. The findings presented confirm recent literature emphasizing that narcissism can be beneficial in some context (Kollmann et al., 2019, reflecting on research by DeNisi, 2015). As such, Maccoby (2000) noted that a positive attribute of narcissism includes the ability to see the bigger picture and utilize their persuasive and charismatic personalities. Moreover, narcissism could be associated with creativity through dynamic social interaction (Kollmann et al., 2019). The findings mentioned support the positive influence on artistic career interests. Contrary, research by Grijalva and Harms (2014) found that narcissistic individuals show a preference toward careers with positions of influence, admiration and power. Which might not be inherent to realistic career interests supporting the findings presented relating to the adverse effect on realistic career interests.

Contrary to expectations, narcissism did not yield a statistically significant effect on career interest or subjective career success. The finding that narcissism does not significantly impact career interest or career success contradicts some of the existing literature (e.g., Grijalva and Harms, 2014; Nevicka et al., 2018), which suggests that narcissistic individuals often succeed in competitive work environments. This might be ascribed to differences in cultural context (e.g., the South African workplace norms) or potential self-perception bias in reporting narcissistic traits. Moreover, earlier research suggesting that narcissists self-promoting inclinations might propel them toward ambitious career goals, affecting both career interest and success (Grijalva and Newman, 2015) which was refuted in the current study. Nonetheless, the findings of the current study indicate that narcissism might not significantly influence career outcomes as initially hypothesized. Furthermore, individuals with narcissistic tendencies might pursue occupations that reinforce inflated self-esteem; however, the lack of substantial interpersonal relationships, along with potential arrogance, could hinder true success (Nevicka et al., 2018).

Lastly, Machiavellianism exhibited a statistically significant positive effect on enterprising career interests. Results presented confirm extant literature in that enterprising career involving leadership, persuasion, and negotiation are desirable to Machiavellians as these roles offer ample opportunities for manipulation and strategizing (Liyanagamage et al., 2022). Similarly, Machiavellianism statistically significantly affected subjective career success. The mentioned research result validates earlier research indicating that although Machiavellianism can produce immediate career advantages through traits of manipulation and calculated tactics, these behaviors ultimately harm sustained career success, as they tend to undermine trust and collaboration (Jonason et al., 2014). Machiavellians might pursue careers that position them in roles that emanate power and influence. However, they rarely achieve subjective career success due to their inability to sustain long-term positive relationships with others (O’Boyle et al., 2012).

While data could not support the hypotheses underscoring the moderating effect of Machiavellianism and narcissism, the moderating analysis offered significant insights into how psychopathy moderates the nexus between investigative career interests and subjective career success. *Per se*, psychopathy weakened the association between investigative career interests and career success, underscoring how psychopathic characteristics hinder individuals from converting their career interests into perceived career success (see, for example, Smith and Lilienfeld, 2013). Individuals possessing strong career ambitions and psychopathic traits might pursue positions that seemingly correspond with innate career interests, such as being in entrepreneurial or competitive milieus. However, their antisocial, apathy and manipulative behaviors inevitably impede subjective career success (LeBreton et al., 2018). Thus, investigative careers are particularly vulnerable to the negative effects of psychopathy ascribed to ethical judgment, critical thinking and independence required in investigative career roles. Impulsivity, lack of empathy, and manipulativeness associated with psychopathy undermine these essential skills, leading to decreased career interest and success in investigative fields. According to this research, the destructive behaviors of those with psychopathic traits can hinder their success in careers aligned with innate occupational interests. In practical terms, psychopathic traits serve as obstacles to achieving career success even though there might be an ideal job position fit (Steinert et al., 2021) especially with investigative career interests.

The findings further highlight the complexities of the Dark Triad traits within the contexts of work, specifically how psychopathic traits disrupt the alignment between career interests and subjective career success. Organizations can prevent potential risks and develop streamlined strategies for dealing with the mentioned characteristics by promptly detecting individuals with elevated levels of psychopathy and Machiavellianism. Furthermore, leadership development and career counseling can benefit from an awareness of how these traits can present in different sectors and job levels, particularly in South Africa, where cultural and social diversity shapes the nature of the working environment (Zettler et al., 2011). The practical implication of the research reported on should be extended to leadership development programs for individuals with moderate Dark Triad tendencies. Interventions for mitigating the negative effects of Dark Triad employees in collaborative settings should be explored in addition to HR policies for detecting and managing high-risk personality traits in organizational hierarchies.

## 8 Limitations and future research

One of the limitations of the research study was the dependence on self-report measures, which are often susceptible to biases such as social desirability or self-promotion, especially when evaluating traits associated with the Dark Triad, where participants might want to present themselves in a positive light (Chaudhary, 2019). Therefore, the accuracy of the results might be influenced when participants underreport or overstate specific characteristics or experiences within the work context. Moreover, the exclusive use of self-reported measures introduces common method bias. Although the study conducted an exploratory factor analysis (EFA) to control for common method bias, other tests such as Harman’s single-factor test, or marker variable techniques could be employed in future studies. A further constraint is the non-probability sampling technique that was used which could have had a negative influence on the external validity of the results. Hence, caution is advised when interpreting the findings. Furthermore, the composition of the study sample could be a further constraint. Although the sample consisted of participants from various industries across a variety of job levels potential sample biases (viz. overrepresentation of specific industries or underrepresentation of vulnerable work populations) could be a caveat of the research reported on. Furthermore, the study did not consider the potential impact of job- related or industry-specific differences on the manifestation of the Dark Triad traits, which could significantly affect how these characteristics relate to career success. To gain a more detailed understanding of the effects of the dark triad traits, future research should investigate these interactions with specific organizational contexts and job roles.

Several recommendations for future research are suggested considering the findings and limitations of the research study. Firstly, future research should explore how different job levels and work sectors could potentially alter the relationship between dark triad traits and career success. Thus, future research studies could ascertain whether the moderation effect vary by industry, job level, or demographic subgroup (e.g., gender differences in Dark Triad expression). This would allow for a more nuanced understanding of the circumstances in which these attributes might be more prevalent or even tolerated. Additionally, given the distinct socio-cultural context of South Africa, future research could benefit from using a mixed- method approach by including qualitative interviews, which could offer deeper insights into how these traits manifest in actual organizational settings. Future research endeavors could potentially investigate the nexus between narcissism, career interests and objective career success. Finally, the effects of the Dark Triad traits on career development and success over an extended period should be monitored by employing a longitudinal study, particularly in leadership positions where the effects on the organizational outcomes might be more significant.

## 9 Conclusion

The research underscores the complex nature of the traits mentioned and the effect thereof on career outcomes. This study found that psychopathy impedes the nexus between career interest and career success specifically for investigative career interests. Considering the direct effects, Machiavellianism negatively influences career success, consistent with previous empirical research findings. While narcissism had a statistically significant influence on realistic and artistic career interests. Psychopathy additionally reverted a statistically significant direct effect on various career interest facets. Finally, subsequent research should focus on contextualizing these results with specific work sectors and using more rigorous research methodologies to evaluate the long-term impact of these traits on career outcomes.

## Data Availability

The datasets presented in this article are not readily available because the dataset is part of a magister study and thus the intellectual property of the University of Johannesburg. Requests to access the datasets should be directed to petronellaj@uj.ac.za.
